# Carcinoid heart disease revealed by cyanosis with both right and left valvular involvement: a case report

**DOI:** 10.1186/s13256-018-1574-6

**Published:** 2018-01-31

**Authors:** Khadija Khay, Salim Arous, Tarik Bentaoune, Abdenasser Drighil, Rachida Habbal

**Affiliations:** Department of Cardiology, Ibn Rushd University Hospital, Casablanca, Morocco

**Keywords:** Carcinoid syndrome, Fibrosis, Carcinoid tumors, Cardiac surgery

## Abstract

**Background:**

Carcinoid heart disease is a frequent complication of carcinoid syndrome. It is related to the release by the carcinoid tumor and/or its metastases of bioactive substances such as serotonin. It is characterized by right-sided valvular involvement and can lead to right-sided heart failure. It is a prognostic factor of carcinoid syndrome.

**Case presentation:**

We report the case of a 53-year-old African woman with an endocrine tumor of her small intestine complicated by carcinoid heart disease, revealed by right-sided heart failure. The diagnosis was confirmed by transthoracic echocardiography, which showed a severe tricuspid regurgitation with a patent foramen ovale, and by increased serum chromogranin A and urinary 5-hydroxyindoleacetic acid.

Initially she was treated with medical therapy (furosemide and injection of somatostatin). Afterwards she was proposed for surgery. The evolution of her treatment was good.

**Conclusions:**

Carcinoid syndrome is complicated in 60% of the cases of a heart disease, and is responsible for an important morbidity and mortality. The prognosis of patients with carcinoid heart disease has improved in recent years through somatostatin analogues and the cardiac surgery.

## Background

Well-differentiated neuroendocrine tumors are rare. They are characterized by the secretion of a vasoactive substance (serotonin), responsible for the appearance of carcinoid syndrome symptoms (flushing, diarrhea, and bronchoconstriction) [1, 2]. The direct action of these vasoactive substances on the heart leads to carcinoid heart disease (CaHD; Hedinger’s syndrome), which is a frequent complication of carcinoid syndrome; CaHD can be the first presentation of carcinoid syndrome in up to 20% of patients. Right-sided heart failure is a prognostic factor of carcinoid syndrome [3].

The management of CaHD is based on medical therapy (somatostatin analogues), resection of the primary tumor, and cardiac interventions. Surgical valve replacement is an effective treatment option for symptomatic patients, since it improves the symptoms and life quality [4].

Through the case of a 53-year-old woman with an endocrine tumor complicated by CaHD, we will discuss carcinoid syndrome and the peculiarities of cardiac involvement. In particular, in this case, we noted the involvement of both the left-sided and right-sided heart, which is rare during carcinoid syndrome. This is related to the presence of a communication between the right and left cavities of her heart, which explains her severe hypoxia and cyanosis.

## Case presentation

A 53-year-old African woman presented with heart failure with increasing dyspnea, lower limb edema, and cyanosis of her lips and extremities with refractory hypoxia. We noted that she did not have a history of heart disease or digestive pathology. She was never operated on previously, and had no history of medical pathology or surgery in her family.

A clinical examination revealed blood pressure of 120/70 mmHg, oxygen saturation at 85%, and a 3/6 diastolic murmur of aortic insufficiency with a holosystolic murmur (grade 4/6) suggestive of tricuspid valve regurgitation. Lower limb edema up to her knee joint was also present. We also detected a flushing with hepatalgia and asthenia. A neurological examination was normal; in particular, there was no sensory or motor deficit and no language disorder. The rest of the clinical examination was also normal.

**Fig. 1:**
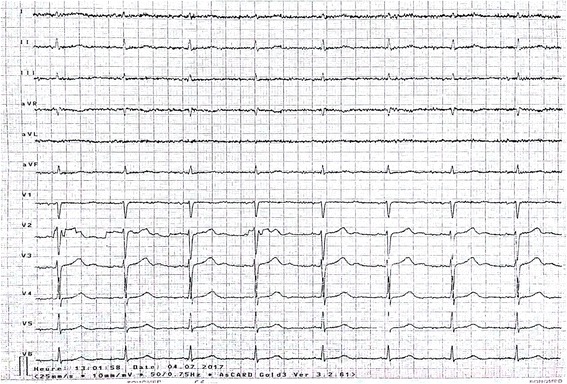
An electrocardiogram of our patient showing no abnormality

A 12-lead electrocardiogram (ECG) showed sinus rhythm and a heart rate of 66 beats per minute (bpm; Fig. 1).

**Fig. 2:**
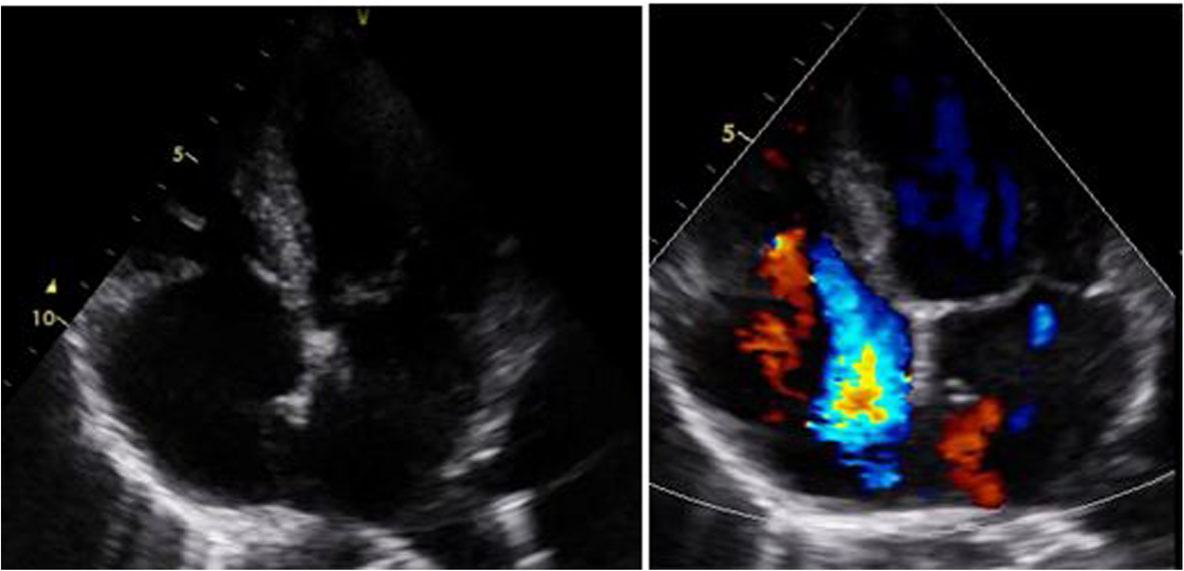
Transthoracic echocardiogram apical four-chamber view showed thickened and retracted tricuspid valves with significant regurgitation and patent foramen ovale

**Fig. 3:**
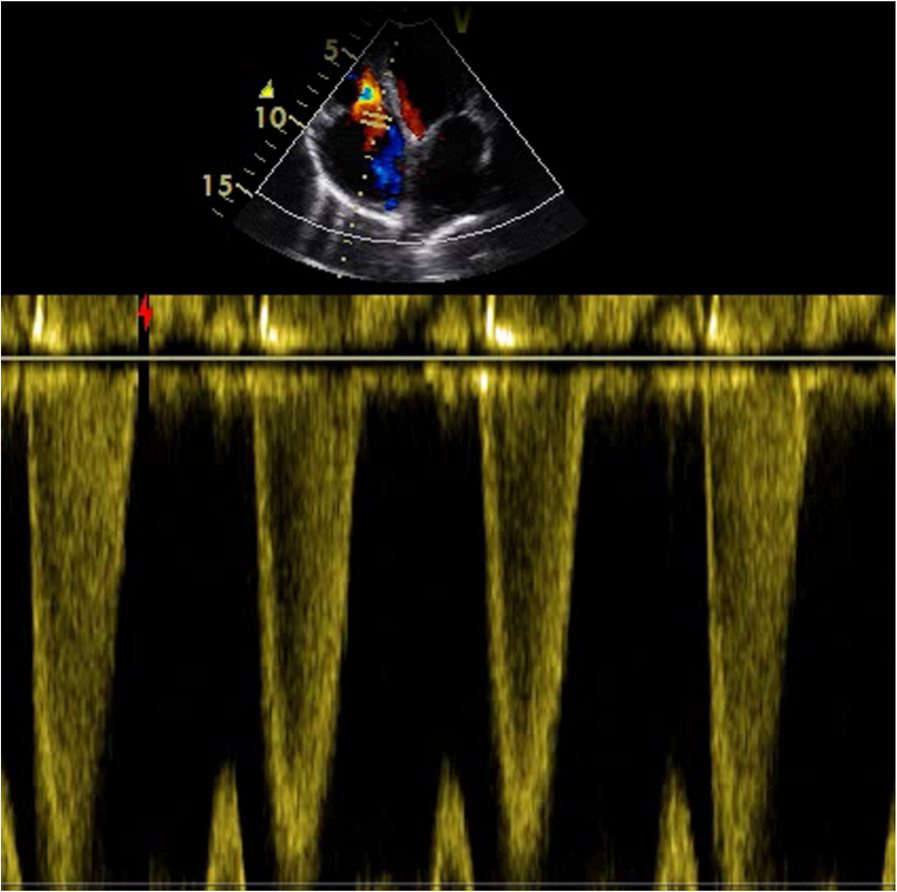
Doppler interrogation of tricuspid regurgitation showing laminar tricuspid insufficiency

She underwent transthoracic echocardiography, which showed slightly dilated right heart cavities with right ventricular end diastolic diameter (RVEDD)/left ventricular end diastolic diameter (LVEDD) of 0.8, right atrial (RA) area of 20 cm^2^, and tricuspid ring of 39 mm with a preserved function in which tricuspid annular plane systolic excursion (TAPSE) was 16 mm and right ventricular (RV) velocity (S’) = 10 cm/second. Tricuspid valves appeared thickened and retracted with restricted mobility and poor coaptation responsible for a severe tricuspid regurgitation (Figs. 2 and 3). Pulmonary valves were thickened with a moderate pulmonary (IP) insufficiency.

**Fig. 4:**
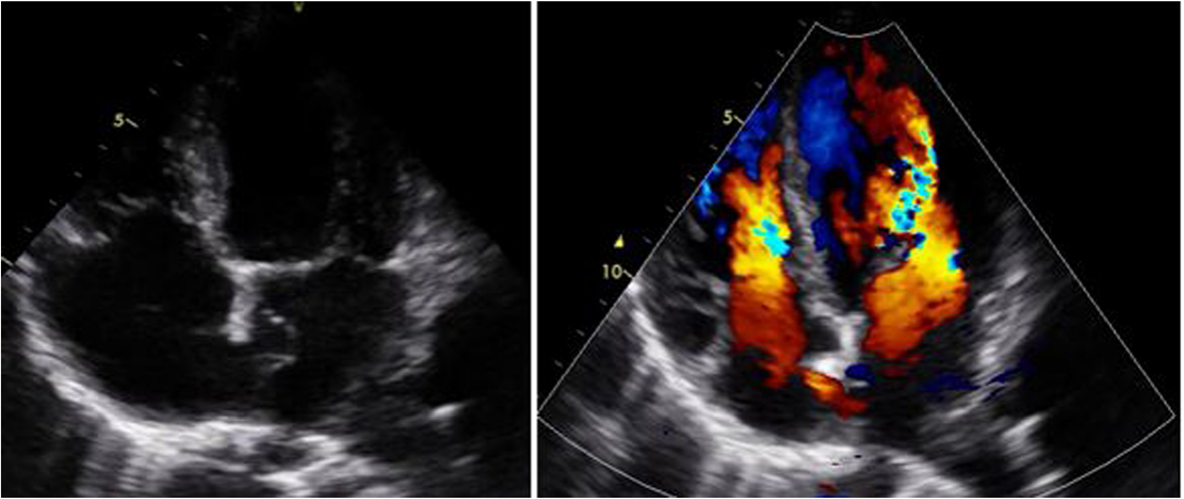
Patent foramen ovale with continuous shunting

A large patent foramen ovale (PFO) with aneurysm of her interatrial septum was detected with continuous right-to-left shunting (Fig. 4).

**Fig. 5:**
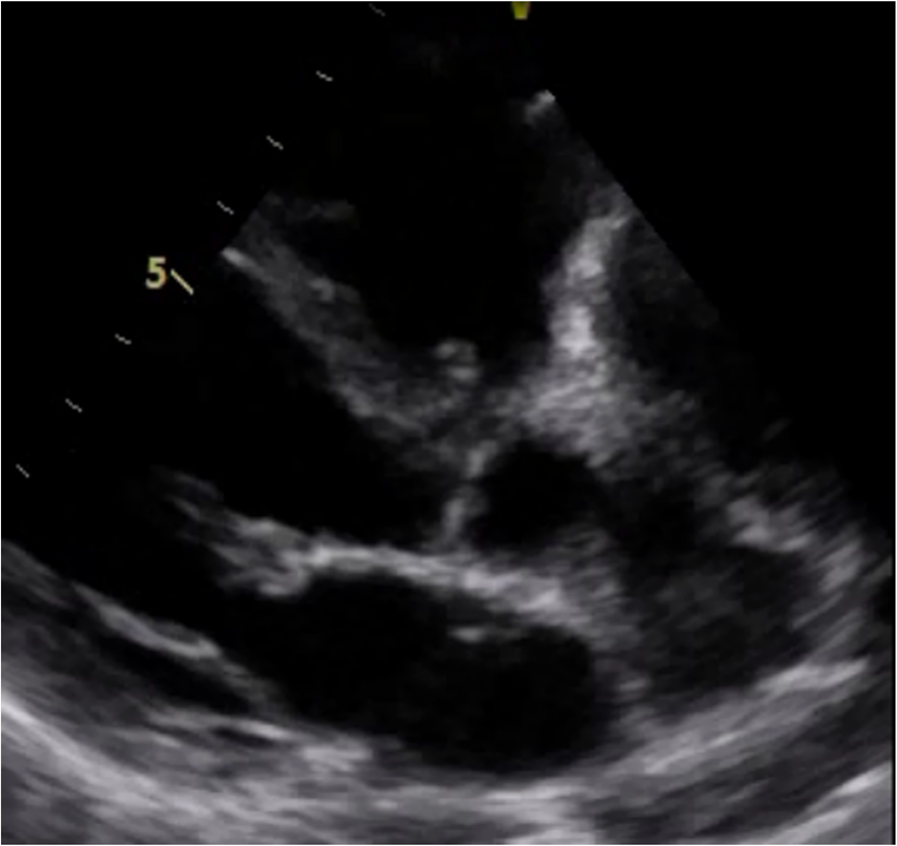
Parasternal long-axis view showed thickened left-sided valves

The left-sided valves were not normal (Fig. 5). Our patient had a moderate aortic regurgitation and minimal mitral insufficiency. The function of the left ventricle was good with ejection fraction (EF) of 55%.

She was initially treated with furosemide. The evolution of her treatment was marked by regression of the signs of her heart failure.

Because of her hepatalgia, a computed tomography (CT) of her abdomen was done and showed multiple hepatic and mesenteric nodules. A resection of 30 cm of her small bowel was done with an anterior gastrointestinal anastomosis.

**Fig. 6:**
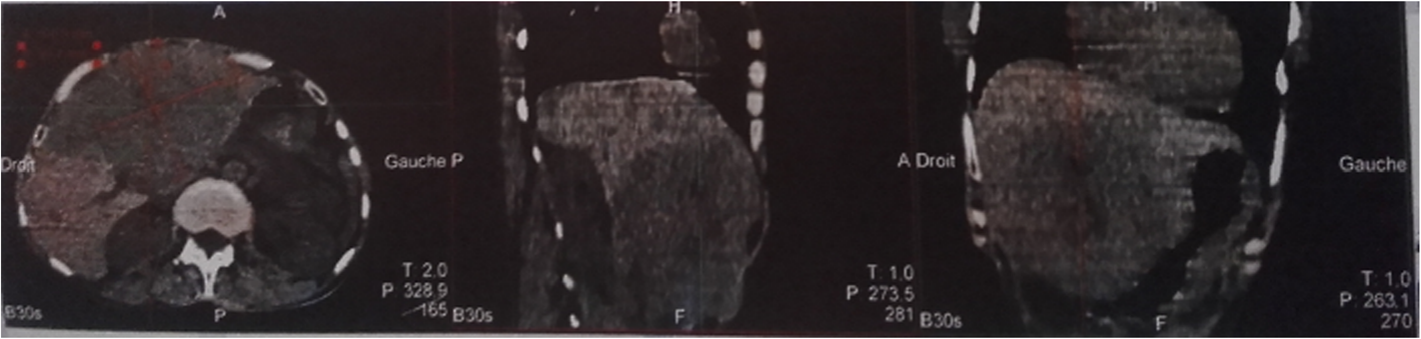
Octreotide scan showing two areas of radiotracer accumulation

Biopsies of her liver and mesenteric nodules showed a secondary mesenteric and hepatic localization of a well-differentiated endocrine carcinoma. An immunohistochemical study showed positivity for chromogranin and negativity for synaptophysin. Laboratory findings revealed increased serum chromogranin A (574 ng/ml, normal < 102 ng/ml) and urinary 5-hydroxyindoleacetic acid (5-HIAA; 1183 μmol/24hours, normal < 40 μmol/24hours). An octreotide scan showed an intense radiotracer accumulation in her liver as well as normal distribution of the radiotracer in her kidneys and spleen (Fig. 6).

Because of all these elements, we concluded that our patient had carcinoid syndrome complicated by the involvement of both the right side and left side of her heart. Her cyanosis was due to a right-to-left cardiac shunt.

Our patient received somatostatin analogue therapy (one injection every 3 weeks). Her 5-HIAA and chromogranin A levels decreased to 450 μmol/24hours and 109 ng/ml respectively. For the heart disease, cardiac surgery was indicated: foramen ovale closure with tricuspid valve plasty.

The evolution of her treatment was marked 6 months after the surgical procedure by the regression of hypoxia with good clinical improvement. An echocardiography control showed minimal tricuspid regurgitation, without residual shunt of patent foramen oval.

## Discussion

Carcinoid syndrome is a paraneoplastic syndrome mediated by humoral factors released by some carcinoid tumors. The majority of neuroendocrine tumors arise from the small intestine, particularly in the ileum, and they release into the systemic circulation a variety of vasoactive substances: serotonin, 5-hydroxytryptamine, 5-hydroxytryptophan, histamine, tachykinins, bradykinin, and prostaglandins, which explain the clinical manifestations of carcinoid syndrome [2].

Carcinoid syndrome is rare and once it is developed, more than 50% of the patients develop CaHD that can inaugurate this syndrome in 20% of cases. It is associated with a poor prognosis [3].

Symptoms of carcinoid syndrome (facial flushing, hypermotility of the gastrointestinal system, bronchoconstriction, and hypotension) usually occur in patients with hepatic metastatic lesions, due to the lack of hepatic inactivation of these released hormones [5].

These vasoactive substances act directly on the valvular endocardium and activate a fibrotic process. Histopathology reveals fibrous plaque-like and endocardial thickening leading to thickening and retraction of the valves. In this disease, the involvement of the right-sided heart valves is predominant and may be in the form of regurgitation and/or stenosis [6, 7]. Right-sided heart failure remains a major factor of morbidity in patients with CaHD [8]. Left-sided cardiac involvement is rarely observed (<10% of patients), particularly in the case of right-to-left shunt (a PFO) or bronchial carcinoids due to the bypassing of inactivation of serotonin within the lung [9].

The diagnosis is essentially based on biological examinations and echocardiography which remains the principal imaging modality in assessment of CaHD [6–10]. Biological examinations are helpful in the diagnosis of CaHD. High levels of N-terminal pro-brain natriuretic peptide (NT-proBNP), chromogranin-A (a neuroendocrine secretory protein), and urinary 5-HIAA (a metabolite of serotonin) are correlated with the progression of CaHD [11, 12]. Echocardiography shows typical valvular involvement: the tricuspid valve is constantly thickened, rigid, and retracted with restricted mobility and poor coaptation which lead to tricuspid regurgitation. Stenosing character is rarer (25% of cases) but may be associated. The pulmonary valve, which is more difficult to study, is reached in 30% of cases with regurgitation or pulmonary stenosis [10–13].

The right cavities and the inferior vena cava are dilated with or without right ventricular dysfunction [14]. Myocardial strain allows detection of an early right ventricular dysfunction in patients with CaHD independently of valvular involvement [15].

Of all patients with CaHD, ≤ 10% have lesions of the left-side valve. CaHD in the left side of the heart is less severe than in the right side of the heart. Left-sided heart involvement in carcinoid syndrome happens in patients with persistent foramen ovale or bronchial carcinoid [16].

Patients with CaHD have a decreased life expectancy compared with patients without cardiac lesions [17]. The treatment of patients with carcinoid syndrome is complex and involves multidisciplinary management. It is based on medical therapy, surgery of the tumor, and cardiac surgery [2].

Medical management consists of controlling the heart failure (diuretics agents and aldosterone antagonist) and symptoms of carcinoid syndrome. Therapy by somatostatin analogues allows improvement of symptoms and quality of life and decreases the incidence of CaHD from 50 to 20% [4].

New agents (telotristat and pasireotide) have shown promising results in patients with carcinoid syndrome refractory to somatostatin analogues [18]. Telotristat etiprate is a potent inhibitor of the synthesis of serotonin. The phase III TELESTAR clinical trial has shown that Telotristat may control the bowel movements in patients with carcinoid syndrome. It represents a new option for the treatment of patients with refractory carcinoid syndrome. However, other research is required to verify the safety and the benefit to control symptoms of that new drug [19].

Interferon alpha can be used as a complementary treatment for somatostatin analogues in refractory carcinoid syndrome. Because of its side effects, it should be initiated by 3 MU thrice weekly and then an individual titration [20].

Surgical resection of the primary tumor and resection of liver metastases appear to decrease the cardiac progression in CaHD and improve prognosis [16]. Since hepatic surgery exposes a patient to the risk of perioperative bleeding, hepatic intra-arterial treatment (transarterial chemoembolization, selective internal radiotherapy) is well suited to patients with hepatic metastases [21].

Valve replacement surgery or valvuloplasty is the only effective treatment for symptomatic CaHD; it improves the symptoms and increases the life expectancy of these patients [22]. The optimal moment for valve replacement surgery is not established. However, valvular surgery is proposed when patients become symptomatic or develop ventricular dysfunction provided they have a life expectancy of at least 1 year [20]. Bioprosthetic valves are generally preferred over a mechanical prosthesis, which requires anticoagulation for life and then exposes patients with liver metastases to the risk of bleeding in addition to the risk of prosthesis thrombosis in tricuspid position [23]. Percutaneous valve implantation is a novel option in high-risk patients with severe CaHD, who have poor performance status and comorbidities that do not allow open surgery [24].

The prognosis of patients with CaHD has improved in recent years through cardiac surgery. In the Mayo Clinic, a retrospective analysis of 200 patients with CaHD found that cardiac surgery improves the prognosis and reduced the mortality related to that disease [25, 26].

## Conclusions

Carcinoid syndrome is rare and can lead to heart failure, which then interferes with the quality of life of these patients. Echocardiography is recommended to evaluate the cardiac involvement in patients with carcinoid syndrome. Surgical valve replacement can improve the clinical outcome and the prognosis of patients with CaHD.
